# Does Heart Rate Variability Predict Impairment of Operational Performance in Divers?

**DOI:** 10.3390/s24237726

**Published:** 2024-12-03

**Authors:** John Freiberger, Bruce Derrick, Ki H. Chon, Md Billal Hossain, Hugo F. Posada-Quintero, Mary Cooter, Richard Moon

**Affiliations:** 1 Department of Anesthesiology, Duke University, Durham, NC 27708, USA; jakefreiberger@gmail.com (J.F.); bruce.derrick@duke.edu (B.D.); richard.moon@duke.edu (R.M.); 2 Department of Emergency Medicine, Duke University, Durham, NC 27708, USA; 3 Department of Biomedical Engineering, University of Connecticut, Storrs, CT 06269, USA; md.b.hossain@uconn.edu; 4 School of Medicine, Duke University, Durham, NC 27710, USA; mary.cooter@duke.edu

**Keywords:** heart rate variability, diving, narcosis, nitrogen, carbon dioxide, oxygen toxicity, cognitive testing, cognitive impairment, MATB-II, anesthesiology, mission, hypercapnia, hyperoxia, underwater breathing, flight simulator, inert gas, undersea, navy, rapture of the deep, random forest, support vector machine, k-nearest neighbor classifier, SMOTE

## Abstract

We examined data from Naval Sea Systems Command grant project N0463A-12-C-001, “Hypercapnia: cognitive effects and monitoring”, with the objective of validating or repudiating heart rate variability (HRV) as a warning sign of cognitive impairment from diving gas narcosis or oxygen toxicity. We compared HRV feature scores to their temporally corresponding cognitive outcomes under normal and narcotizing conditions to identify specific HRV features associated with cognitive changes. N0463A-12-C-001 was conducted between 17 September 2013 and 29 January 2016 and employed NASA’s multi-attribute task battery (MATB-II) flight simulator to examine the independent effects of CO_2_, N_2_, and O_2_ partial pressure on diver performance at simulated depths up to 61 msw (200 fsw). We assessed the association of 23 distinct HRV features scores from 432 of the study’s analyzable exposure stages in relation to MATB-II’s four performance subclasses (motor, memory, attention, strategy) while controlling for exercise and CO_2_, N_2_, and O_2_ gas partial pressure. Performance decrements were associated with normalized high-frequency HRVfeatures (HFnu, *p* = 0.0016) and the number of pairs of successive R-R intervals that differed by more than 50 ms (NN50count1, *p* = 0.04). Secondary analysis with stratification restricted to non-exercise stages showed that several HRV parameters, including root mean square of the successive difference (RMSSD, *p* = 0.0015), width of Poincaré plot (*p* = 0.0017), NN50count1 (*p* = 0.0019), and standard deviation of normal-to-normal R peaks (*p* = 0.0082), were associated with performance impairment. The RMSSD association retained statistical significance after Bonferroni correction for multiple tests. HRV features collected from divers tested under narcotizing conditions of breathing gas partial pressure and exercise were associated with performance impairment.

## 1. Introduction

Inert gas narcosis, sometimes called nitrogen narcosis, and CNS oxygen toxicity are operational risks for all divers because they adversely affect cognitive performance in the underwater environment. Narcosis is a reversible condition which consists of symptoms such as euphoria, impaired neuromuscular coordination, delays in auditory, visual, and tactile responses [1], diminished memory, and impaired concentration that adversely affects operational performance in divers. It was first reported in the early 19th century by Colladon (1826), Junod (1835), and Green (1861) [2,3,4,5,6,7,8,9], and it remains incompletely understood. Divers experience decreased psychomotor performance when exposed to higher than normal inspired partial pressures of N_2_, CO_2_, other inert gases, and possibly O_2_ [10,11]. High inspired partial pressures of oxygen are dangerous for divers because they cause CNS toxicity, including seizures [11,12,13]. Although several alternatives exist to treat and prevent the risks of gas narcosis [14,15], early detection is the key to minimizing deleterious effects [16].

Previous studies have shown that diving affects the autonomic nervous system (ANS) [17,18,19,20,21,22]. Heart rate variability (HRV) analysis is a widely used noninvasive tool to assess ANS, making it a potentially promising approach to detect gas narcosis. HRV involves calculations that correlate the variability of the time intervals between consecutive heart beats in the electrocardiogram (ECG) with clinical outcomes [23]. Variations in heart rate represent dynamic interactions between parasympathetic and sympathetic nervous activities in the heart, which modulate the oscillations in the intervals (ms) between consecutive heartbeats [22,23,24,25]. The basis of these calculations is routine ECG tracing, a measurement that is easily and non-invasively obtained using standard cardiogram devices. Abnormal HRV was first noted in neonates during the mid-twentieth century [26,27] and has since been associated with post-cardiac infarction mortality [28,29], autonomic neuropathy in diabetic patients [30,31], and changes in cognitive function [32]. Because HRV is a non-invasive approach that provides insights into aberrant neurological activity, it has the potential to provide early warning signs for narcosis and CNS oxygen toxicity in divers. If HRV features are shown to predict, coincide with, or even echo narcosis or oxygen toxicity symptoms, it could be a valuable tool to predict these potentially fatal symptoms in divers.

To the best of our knowledge, no prior studies have assessed HRV in association with diving narcosis. Previous studies using HRV have shown that diving affects the ANS [17,18,19,20,21]. A simple analysis of HRV that could continuously and non-invasively assess the degree of sympathetic and parasympathetic balance in a diver’s autonomic nervous system would provide early warning of dangerous conditions. Previous studies have shown that changes in autonomic activity may predict central nervous system (CNS) oxygen toxicity [33] and that cardio–pulmonary baroreceptor afferent signals can work in the reverse direction (from the periphery to the central system) to modulate brain excitation and influence susceptibility to oxygen toxicity seizures [34]. HRV has been shown to be correlated with scores on tests of global cognitive performance [32,35,36,37].

We examined data from a previous study to test whether heart rate variability (HRV) could provide warning signals to avoid cognitive impairment during a dive [38]. HRV studies have shown that diving affects the autonomic nervous system [9,17,18,19,20,39,40,41] and that changes in autonomic activity may predict CNS oxygen toxicity [33]. If changes in HRV reliably precede the onset of narcosis or oxygen toxicity, then they could be used as measures to predict and potentially avoid dangerous cognitive risks associated with the dive. We extracted several HRV features in both the time domain and frequency domain and analyzed their association with NASA Multi-Attribute Task Battery (MATB) II test scores. Furthermore, we applied machine learning algorithms to classify normal vs. impaired cognitive performance based on HRV parameters [42,43].

## 2. Materials and Methods

This study analyzed data from Duke University Medical Center’s institutional review board’s approved re-review of a previously published trial that examined cognitive effects of potentially narcotizing mixtures of oxygen, nitrogen, and carbon dioxide during simulated dives to depths up to 61 msw (200 fsw) [38]. Between 17 September 2013 and 29 January 2016, 50 subjects selected to meet US Navy age and fitness profiles [44] were tested during 31.5 °C (88.7 °F), head-out, water-immersion exposure in a 10 by 18-foot hyperbaric chamber using NASA’s MATB-II flight simulator [45].

**Figure 1:**
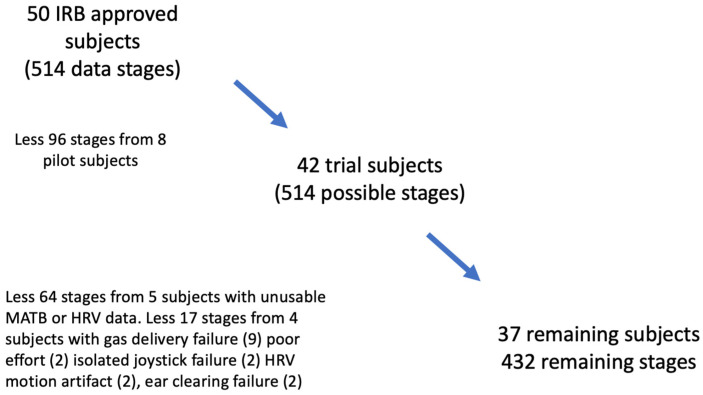
Consort Diagram.

The fifty male volunteers were between 20 and 48 years of age and met US Navy fitness profiles [44]. ECG signals for HRV analysis were collected using 3M Red Dot Ag/AgCl electrodes and a Hewlett-Packard (Palo Alto, CA, USA) M1094B/M1176A monitoring system. The analysis to extract HRV features from the ECG signals is described in detail below. All data were used if adequate “R” wave identification, a control stage, and compliant MATB responses were present. Stages with technical failures and stages from subjects who were not compliant with the experimental protocol were not included in the analysis. Signs of noncompliance included surreptitious breathing around the mouthpiece (confirmed by discordant arterial inspired or end-tidal O_2_ or CO_2_ partial pressure measurements), the strategic use of multiple anticipatory keystrokes (identified from repetitive programmatic keystroke patterns with excessive numbers of “false positive” responses), low effort (evidenced by non-exposure-associated lack of focus on the simulator tasks or repeatedly ignoring requests to not remove the mouthpiece to talk). The consort diagram is shown in Figure 1. After all exclusions, 37 subjects and 432 stages remained.

### 2.1. Consort Diagram

Subjects were randomly assigned to one of four protocols (A–D) that encompassed a total of 20 distinct environmental exposure stages defined by their gas partial pressure and exercise conditions. To increase statistical power, stages with conditions of high N_2_ exposure at PiN_2_ = 446.8 kPa and 565.4 kPa (4.41 and 5.58 ATA) were combined into single exposure group, thereby yielding 16 separate gas and exercise *pooled exposure groups*. Table 1 shows the pooled exposure groups.

ATA, pressure in absolute atmospheres; FiCO_2_, FiN_2_, FiO_2_, fraction of respective breathing gases; PiCO_2_, PiN_2_, PiO_2_, partial pressure of respective breathing gases.

### 2.2. Data Acquisition Methods

Electrocardiograms, radial arterial blood pressure, heart rate, respiratory rate, tidal volume, end-tidal CO_2_, end-tidal O_2_, and end-tidal N_2_ were continuously monitored and digitally captured using ADInstrument’s LabChart 7 Pro software (Colorado Springs, CO, USA). An Apple iPad running FilemakerGo version 11 (https://www.claris.com (accessed on 11 February 2024)) provided a detailed playbook for each individual experiment and recorded the precise timing of each experimental event. A FilemakerPro version 16 database collected, stored, and synchronized each subject’s personal information along with the performance and physiological data from the multiple experimental gas, pressure, and exercise conditions. All devices were synchronized to an internet-obtained time signal.

### 2.3. HRV Analysis

ECG tracings for HRV analysis and physiology data (arterial line tracings, end-tidal gas partial pressures, work, and respiratory rates) were selected for each 5 min experimental stage [31] and saved as separate files using a custom designed macro in LabChart 7. The raw ECG signal was filtered using a variable-frequency complex demodulation (VFCDM)-based denoising method [46]. Baseline drift was removed from the raw ECG using the moving window median filtering technique described by Brennan [47]. R-wave peaks were detected using an empirical method based on adaptive noise decomposition (CEEMDAN) [48]. After automatically detecting the R-wave peaks, tracings were manually reviewed and corrected if needed. An instantaneous HR signal at a sampling rate of 4 Hz was created using the technique described by Berger [48,49]. HR signals were then downsampled to 1 Hz with the mean and low-frequency trends removed. HRV analysis was conducted after a 2 min “gas wash-in” delay following each gas switch. Outcomes were reported as percent change from air breathing at rest baseline measurements. Three classes of HRV calculations were conducted on the HR sequences: (1) time–domain features (TD); (2) power spectral density (PSD) features; and (3) principal dynamic mode features (PDM). A total of 23 distinct HRV features were calculated.

#### 2.3.1. Time-Domain Features (TD)

TD calculations were made from instantaneous R-R intervals. Low frequency (LF) was defined as 0.04–0.15 Hz. High frequency (HF) was defined as 0.15–0.4 Hz. LF represents both sympathetic and parasympathetic tones whereas HF represents the parasympathetic tone [50]. For autoregressive (AR) modeling we used the even-sampled HR sequence [49]. Table 2 lists the fifteen TD HRV features.

#### 2.3.2. Power Spectral Density Features (PSD)

PSD values of the heart rate sequences were calculated using the Welch periodogram method [51]. From the PSD analysis, we calculated the mean spectral power (total psd) in the LF and HF bands, and the LF-to-HF ratio. A 128-point fast Fourier transformation with a frequency resolution of 0.0078 Hz and a Hanning window with 50% overlapping segments was used. In keeping with the recommendation from the HRV task forces [50] we assigned the LF and HF bands to 0.04–0.15 Hz and 0.15–0.4 Hz, respectively. The eight PSD features calculated from the PSD are listed in Table 3**.**

#### 2.3.3. Principal Dynamic Mode Features (PDM)

PDM feature analysis is a nonlinear technique that uses eigenvalue decomposition. In this study, we applied PDM feature analysis to separate dynamic components of the sympathetic and parasympathetic nervous system from within the ECG signal. The two dominant PDM features, PDMsymp and PDMpara, in the LF and HF bands derived from PSD, are considered to correspond to sympathetic and parasympathetic nervous system activity, respectively [52,53]. The major difference between PDM and PSD is that PDM specifically accounts for the inherent nonlinear dynamics of HR control, which PSD cannot. PDM employs the statistical technique of principal components analysis (PCA). PCA selects only the dominant eigenvectors and eigenvalues, as these are closely related to the true characteristics of the signal, since non-dominant eigenvectors and eigenvalues represent noise or nonessential characteristics. A minimum set of basis functions were determined using principal component analysis.

The PDM features were calculated using the Volterra–Wiener kernels based on expansion of Laguerre polynomials [54]. Among all possible choices of expansion bases, some require the minimum number of basis functions to achieve a given mean-square approximation of the system output. This minimum set of basis functions is termed the principal dynamic mode of the nonlinear system. Thus, principal component analysis separates only the essential dynamic characteristics from a signal that is likely to be corrupted by noise and non-system related dynamics. The PDM method requires both the input and output data, but since we had only the output signal of HR recordings, we used the following steps to create an input signal. The aim was to create an input signal with broadband characteristics. We used HR delayed by one unit as the input and undelayed HR as the output signal to obtain the PDMs. We used the first four PDMs to reconstruct the output signal. The justification for using the first four PDMs is because they accounted for our set threshold value of 90% of the HR dynamics. These first four PDMs reflected the dynamics of the sympathetic and parasympathetic systems [21]. PDM outputs were converted to the frequency domain using fast Fourier transform to facilitate interpretation of the two ANS activities; these are usually illustrated in the frequency domain. For this study, we used 8 Laguerre functions with a memory length of 60. The detailed steps involved in the calculation of PDMs as well as for determining the Laguerre functions and the memory lengths were as previously described [55]. The two PDM features are listed in Table 4.

### 2.4. Cognitive Testing Platform (MATB-II)

NASA’s MATB-II was the cognitive testing platform used: a multi-tasking JavaScript flight simulator created in 1992 and revised in 2014 [45]. It has 4 simultaneously administered and independently scored subtasks: motor (TRACKING), attention (MONITOR), memory (COMMUNICATION), and strategy (PUMPS). The sequence of subtask events can be programmed into a single events file that can be reused for each subject, ensuring identical sequence and nearly identical timing of the individual queries for all subjects. Subtask impairment thresholds were assessed at the 15%, 30%, and 45% levels of adverse changes from subject’s air breathing at resting baseline. After a sensitivity analysis, a 30% decrement from baseline was chosen, based on the R^2^ values for the regression lines of the individual subtask scores against the number of impaired subtasks for each stage. An overall impairment score, the *stage impairment score*, was defined as the 5-level (0–4, none to severe) sum of the “impaired” MATB subtasks.

### 2.5. Machine Learning Analysis

**Figure 2:**
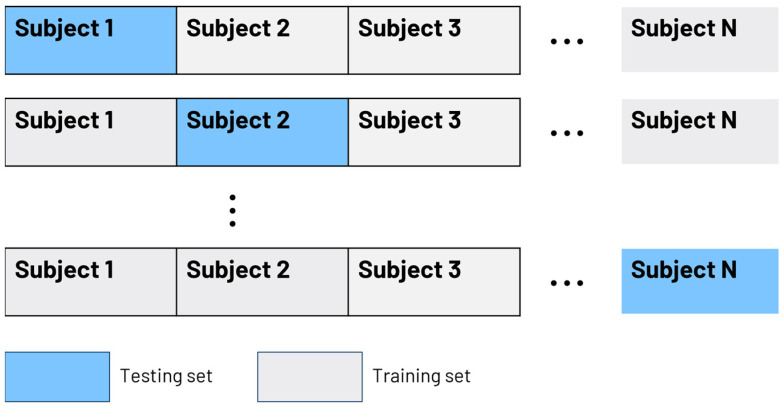
Machine learning LOSO validation scheme.

We used a supervised machine learning algorithm to distinguish between normal and impaired operational performance [56,57]. Fifteen separate HRV features with direct physiological interpretation were selected for machine learning analysis. The input vectors were each diver’s HRV data during every stage of the protocol. The output vectors were class labels (normal or impaired). We tested 4 different machine learning techniques: random forest, support vector machine with radial basis function kernel, linear support vector machine, and k-nearest neighbor classifier. We chose kernel SVM, and random forest [58] as classifiers because of their interpretability. The dataset was split into training and test sets using *leave-one-subject-out (LOSO)* cross-validation in order to learn and create a decision boundary between the two classes. This was equivalent to k-fold cross-validation with k = 37, but the data was split subject-wise instead of sample-wise. In order to perform a subject-independent evaluation, we followed a “leave-one-subject-out” strategy where one subject was left out for testing and the remaining ones were used for training as depicted in the Figure 2. We optimized the model parameters using 10-fold cross validation along with a grid search technique on the training data in the first iteration. The same model parameters were used in the subsequent runs. For the SVM, we varied the parameter C from 0.001 to 1000 with an increment of 10 times the previous value. For KNN, we varied the number of neighbors from 2 to 5 with an increment of 1, and for random forest, we varied the number of estimators (20 to 100) and maximum depth of the trees (3 to 20). Since we had a relatively small dataset, the model parameter ranges were determined appropriately to reduce overfitting. The optimized model parameters were as follows: for KNN, 3 nearest neighbors; for SVM, C = 1; and for random forest, 30 estimators and a maximum depth of 5.

We computed the accuracy and F1 score of the machine learning model for performance evaluation, where accuracy was the measure of all the correctly identified cases. The **F_1_** score was the harmonic mean of the precision and recall, where precision refers to the ratio of the number of true positives and the number of all positives, and recall represents the ratio of the number of true positives and the number of all the samples that should have been identified as positives. We selected the best machine learning model in terms of F1 score. The best machine learning model was then trained using the entire dataset in order to determine the feature importance for that model. Because the data were unbalanced, the most frequent classes (in the training data) were balanced using a recognized upsampling strategy called SMOTE [59]. The predicted class labels were then compared to the assigned (reference) labels from their *stage impairment scores* and evaluated for accuracy and F1 score. We used nonlinear soft margin because it can assign class designations when classes are overlapping [57].

### 2.6. Statistics Employed

All notes and raw data files from N0463A-12-C-000 were reviewed for technical errors before being processed and analyzed for this study. Repeated-measures linear regression identified associations of environmental exposure settings (gas mix, exercise), subject characteristics (age, diving experience, video games experience, stage order), and HRV metrics with cognitive impairment as reported by the *stage impairment score*. Analysis was performed in SAS v 9.4. Summary statistics, t-tests, 95% CI graphs, and correlations were calculated in SPSS V23. *p* < 0.05 was considered significant. Machine learning non-linear regression analysis was performed using a support vector machine with RBF kernel, with the HRV features used to regress the cognitive performance scores. Performance was evaluated using the leave-one-subject-out cross-validation strategy. Classification and interpretation techniques included random forest, support vector machine with radial basis function kernel, linear support vector machine, and k-nearest neighbor classifier [56,57,58,59,60]. For a measure of fitness of the model, we report the average R-squared value and the average root mean squared error (RMSE) of the fitted regressor on the testing samples.

## 3. Results

### 3.1. HRV Feature Scores Were Associated with Performance Impairment

Gas partial pressures and HRV scores were significantly associated with performance impairment. In our initial model, we found independent impacts of high CO_2_, N_2_, and O_2_ partial pressures, but not diver’s age, video games experience, or dive training, on cognitive impairment (Table 5). A subsequent model of main effects and two-or three-way interactions identified an interactive impact of gas mix with exercise, but there were no 2-way interactions between gases without the addition of exercise (Table 6). These results confirmed that exercise, CO_2_, N_2_, and O_2_ partial pressures were strongly and independently associated with performance impairment, as seen in the initial study [38]. When we included HRV metrics in a repeated-measures linear regression that controlled for exercise and gas mix, there were significant associations between HFnu (*p* = 0.025) and LF(*p* = 0.041) and cognitive impairment (the *stage impairment score*) (Table 7). When we explored the potential for interaction effects between the HRV feature scores and *stage impairment scores* we found that HFnu (*p* = 0.0016) and NN50count1 (*p* = 0.0404) had significant interactions, and total_psd (*p* = 0.066) and LFnu (*p* = 0.079) had near-significant interactions with exercise. Therefore, due to the large effect of exercise, we stratified the dataset by exercise. Secondary analysis restricted to non-exercising stages identified RMSSD (*p* = 0.0015), SD1 (*p* = 0.0017), NN50count1 (*p* = 0.0019), NN50count1 (*p* = 0.0049), SDNN (*p* = 0.0082), SD2 (*p* = 0.0228), and AR_1 (*p* = 0.0319) as being significantly associated with cognitive impairment (Table 8). After Bonferroni correction for multiple testing, the association with RMSSD in the non-exercise stages retained statistical significance.

### 3.2. Machine Learning Analyses of the Utility of HRV to Predict Cognitive Impairment in Individual MATB-II Subclasses

Cognitive performance during an individual stage was defined as impaired if it showed a 30% or more adverse change from baseline performance. Moreover, since there were four different cognitive tasks, we calculated an overall impairment score depending on how many of these tasks showed impaired performance. Thus, a subject could score from 0 to 4 (where 0 meant no impaired performance at all and 4 meant all four cognitive performance metrics were impaired) for the combined four stages. The dataset contained 420 samples from 34 different subjects. In order to find the best association between HRV features and the cognitive performance scores, we tested four different machine learning algorithms (namely, random forest, support vector machine (SVM) with radial basis function (RBF) kernel, linear SVM, and k-nearest neighbor classifier (KNN)) for classification of the different impairment scores and regression of impairment scores from HRV features. We first used machine learning classifiers to differentiate between normal and impaired performance for each of the MATB subtasks. Table 9 shows accuracies and F1 scores of non-stratified machine learning classification assignments by MATB subtask.

**Figure 3:**
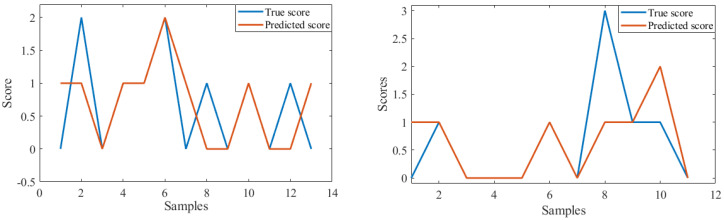
Prediction of performance score using regression.

To further test the utility of the machine-learning algorithms for making class assignments, we performed nonlinear regression using a support vector machine algorithm with RBF kernel whereby the HRV features were regressed against the *stage impairment score (0–4)*. The regression performance was evaluated using the leave-one-subject-out cross-validation strategy. We calculated the average R-squared value, a measure of fitness of the model, and the average root mean squared error (RMSE) of the fitted regressor on the testing samples (Table 10). Figure 3 is a graph that shows two examples of the machine-learning predicted output (orange line) and corresponding observed scores for the MATB subtasks (blue line). The figures indicate how the regression prediction followed the true score, with a decent overlap between the predicted and the actual score; note the complete overlap in scores for the MATB subtasks between true and predicted values for samples 3–6 in the left panel and 2–7 in the right panel of Figure 3). When machine-learning techniques were employed, HRV feature scores predicted *stage impairment scores* at accuracies around 80% for all MATB subclasses.

### 3.3. Use of Machine Learning to Address Confounding by Exercise

While the multiclass classification and the regression analysis showed promising association of HRV features with cognitive impairment, confounding by exercise led us to stratify and analyze the exercise and resting stages separately. Machine learning classifications for resting and exercise stages were generated independently. Out of the total 420 examples, 230 examples were collected during resting and 190 examples were collected during exercise. Since the number of samples was highly reduced after separating resting and exercise stages, we defined combined scores of 1 to 4 as one class (impaired) and scores of 0 indicated non-impaired performance. Then, we performed machine learning classification separately for the resting and exercise stages. The performance of the classification process is reported in Table 11. Machine learning analysis performed slightly better using the exercise strata of the HRV feature scores than with the non-exercise model. The reason behind the poor classification performance with the resting-stage data was the imbalanced impairment of cognitive performance, as most subjects had impairment score of 1, a few had a scores of 2, and there were no scores of 3 or 4. Hence, the impairment during resting stage was largely represented by the score of 1, considered mild impairment in the range from 1–4. In addition, an impairment score of 1 indicated that only one of out the four subtasks was not completed correctly; hence, there might not have been much difference between normal and impaired in these cases. In contrast, the classification during the exercise stages showed more promising results. Since all the training and testing example data were acquired during exercise, we can ignore the effect of exercise among them. Moreover, the impairment scores were not as imbalanced as in the resting case; the scores were more distributed between 1–4 albeit, there were more impairment scores of 1 or 2 than 3 or 4. Thus, given these more balanced impairment scores, the HRV features were more effective in classifying impaired performance, which supports association of HRV features with cognitive performance.

## 4. Discussion

### 4.1. HRV as a Predictor of Diving-Related Cognitive Impairment

This study shows that HRV during 3.5 min of analysis was associated with impaired cognition in divers under narcotizing conditions. When using repeated-measures linear regression, the association was stronger in the non-exercising strata of the data. Machine-learning techniques demonstrated reasonable predication accuracy (Table 7) even when applied to the exercise strata of the data.

### 4.2. HRV as an Early Warning of Diving Narcosis

Although some HRV features were associated with cognitive impairment, the utility of HRV as an early warning signal remains unproven and deserves further study. We established a 90 s “gas wash-in” time before beginning HRV sampling; therefore, the sampling intervals for the 5 min stages were only 210 s in duration, probably an imprecise period for temporal resolution to provide a useful warning signal. Therefore, the answer to the practical question of whether a diver’s autonomic state precedes, coincides with, or echoes narcosis-induced cognitive impairment is likely to require longer sampling periods.

### 4.3. HRV as an Autonomic Signal in Diving

**Figure 4:**
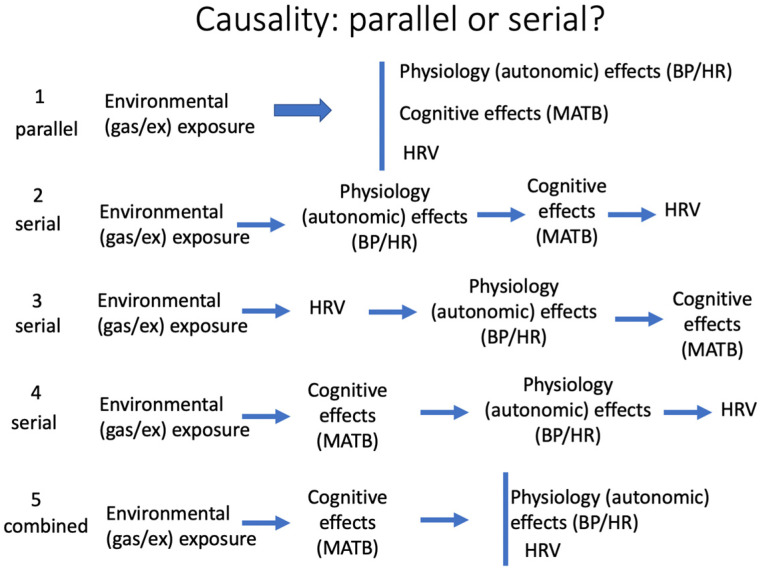
Chain of causality.

Although these findings do not have sufficient temporal resolution to function as an early warning system for narcosis, they may provide insight into the causal relationships that exist between the autonomic nervous system and cognition. The effects of the immersed-diving environment and narcotic gases on cognition and the ANS are entwined. The ANS is influenced by immersion and by changes in O_2_ partial pressure [20,34,40,61,62,63,64,65]. Narcotizing gases influence BP and HR [38] and immersion alone has been hypothesized as a cause of cognitive impairment [66,67]. There are known associations between executive function and HRV [32,68]. However, we do not know whether HRV directly reflects current ANS status or whether it is a link in the chain of causality, transmitting signals generated by environmental conditions (gas and exercise state) to autonomic centers in the brainstem. Figure 4 summarizes five of many possible theoretical options that are consistent with long-standing theories of diving narcosis [13]. These models all begin with the assumption that a diver’s environmental exposure drives downstream effects on physiology, cognition, and presumably, HRV. The downstream effects could be independent of each other (Figure 4, parallel model 1) or possibly subsequent to other inputs (Figure 4, serial and combined models 2–5). Models 4 and 5 assume a primary role for the environmental state with a subsequent upstream role for the traditional narcosis-driven cognitive changes that generate downstream responses in BP, HR, and HRV. These models are supported by our findings that HRV feature scores were actually better correlated with divers’ perception of impairment than with actual impairment itself (Table 12).

Divers’ own perceptions of their cognitive impairment are known to be poorly correlated with actual measurements made by unbiased and independent observers [38,69]. Therefore, if HRV is a narcosis-driven autonomic response to environmental conditions, then, model 5 would be the parsimonious choice. However, if model 5 is correct, then warning changes in HRV would be most likely to occur too late to usefully warn of impending cognitive impairment. Moreover, if perception of cognitive impairment, existent or not, drives autonomic responses, an HRV-based alarm could trap a diver in a recursive loop in which stressful emotions, like stage-fright, generate, where stressful emotions generate autonomic responses triggering cognition alarms which then dangerously amplify the warning circuit. Further study is needed.

### 4.4. Machine Learning to Predict Cognitive Performance Using HRV Scores

Note that while the cognitive performance impairment classification results for the resting stages were poor (F1 score around 40%), the results for the exercise stages were relatively accurate. The reason behind such poor classification performance during the resting stages was the imbalance that characterized the cognitive performance impairment scores during the resting stages, as almost all of the impaired stages during resting had an impairment score of 1, very few had 2, and there was no score of 3 or 4. Therefore, it is likely that a score of 1 during resting did not properly represent impairment, since some of the subjects could have neglected one particular task to score higher on other tasks.

In contrast, the classifications of exercise stages showed some promising results. Since all the training and testing examples were from during exercise, we can ignore the effect of exercise among them. Thus, the results demonstrate that these HRV features were effective in classifying impaired performance, which confirms a significant association of HRV features with cognitive performance. It is logical that most of the impaired performance happened during exercise. However, this result shows that HRV features were able to distinguish the impaired and non-impaired exercise examples with significant accuracy. If we are able to identify whether the subject is resting or exercising, we can deploy classifiers of cognitive performance impairment tailored to the type of physical activity, and higher accuracy can be achieved.

The machine learning predictions of cognitive performance using the HRV scores showed promising and moderate success. However, because of technical shortcomings with the data, we believe this area requires further study. A few limitations of this study included the short ECG length (3 min, whereas more than 5 min for HRV analysis is recommended in the literature), low ECG sampling frequency (100 Hz, whereas a minimum of 250 Hz is recommended [23]), ECG problems (motion artifacts, respiratory artifacts), and loss of cognitive data from subjects who did not finish some of the stages. Moreover, the relatively small cognitive performance dataset is insufficient to reach a definitive conclusion about the usability of HRV for predicting divers’ risk of narcotizing environmental exposure. Therefore, we believe further study is required in this area, with a better and relatively larger dataset. We believe HRV should be supplemented by other measurements designed to assess autonomic condition, such as electrodermal activity (also known as galvanic skin response) [70,71].

## 5. Conclusions

Because of its simplicity and wide usage, HRV is an attractive technique to assess divers’ autonomic state. The experience gained from this study should be helpful for later projects. Moreover, the N0463A-12-C-000 dataset will remain an import resource for further investigations. The main finding of this study is that we identified several HRV parameters that were associated with performance impairment, and that machine learning models were able to predict diving-related cognitive impairment with reasonable accuracy via the use of HRV parameters. Although HRV features can partially predict cognitive impairment, we conclude that HRV should be supplemented by other techniques designed to assess autonomic condition, such as electrodermal activity (also known as galvanic skin response) [70]. The MATB flight simulator remains a useful tool to assess performance in a multi-tasking environment, but a more modern multi-tasking program that combines the ease of administration of the MATB-II with simple-to-score, mission-relevant subtasks would be very useful for future investigators. Longer measurement times and a careful search for symptoms of impairment or incipient O_2_ toxicity are underway, with an ongoing NAVSEA-funded study at Duke University. This study also aims to associate autonomic function with neurological function, employing the MATB. The work may also answer remaining questions about incipient O_2_ toxicity and the nature and validity of the concept of O_2_ narcosis.

### Study Limitations

Methodological problems included (1) ECG motion and respiratory artifacts, (2) absence of cognitive data from subjects too narcotized to complete their assigned stage assessments; and (3) no accounting for mechanoreceptors and ventilatory response; meanwhile, (4) baroreceptor sensitivity analysis and other early warning assessments were not built into the study from the beginning; and (5) longer stage times or longer time without change in gas or exercise state are recommended.

## Figures and Tables

**Table 1:** Pooled exposure groups.

Pooled Exposure Group (*N* = 16)	ATA	Kpa	FiCO_2_	PiCO_2_ ATA	FiN_2_	PiN_2__ATA	FiO_2_	PiO_2__ATA	Exercise
01_air	1	101.3	0.00%	0	79.00%	0.79	21.00%	0.21	0
		101.3							0
02_CO_2_	1	101.3	7.50%	0.1	71.50%	0.72	21.00%	0.21	0
03_O_2_	1	101.3	0.00%	0	0.00%	0	100.00%	1	0
04_CO_2_O_2_	1	101.3	7.50%	0.1	0.00%	0	92.50%	0.93	0
05_N_2_	4.7	476.2	0.00%	0	95.50%	4.49	4.50%	0.21	0
	5.8	587.7	0.00%		96.40%	5.58	3.60%		
06_N_2_O_2_	5.8	587.7	0.00%	0	78.00%	4.51	21.00%	1.22	0
07_CO_2_N_2_	4.7	476.2	1.60%	0.1	93.90%	4.41	4.50%	0.21	0
	5.8	587.7	1.30%		95.10%	5.5	3.60%		
08_CO_2_N_2_O_2_	5.8	587.7	1.30%	0.1	77.70%	4.5	21.00%	1.22	0
09_ex_air	1	101.3	0.00%	0	78.00%	0.78	21.00%	0.21	1
10_ex_CO_2_	1	101.3	7.50%	0.1	71.50%	0.72	21.00%	0.21	1
11_ex_O_2_	1	101.3	0.00%	0	0.00%	0	100.00%	1	1
12_ex_CO_2_O_2_	1	101.3	7.50%	0.1	0.00%	0	92.50%	0.93	1
13_ex_N_2_	4.7	476.2	0.00%	0	95.50%	4.49	4.50%	0.21	1
	5.8	587.7	0.00%			5.58	3.60%		
14_ex_N_2_O_2_	5.8	587.7	0.00%	0	78.00%	4.51	21.00%	1.22	1
15_ex_CO_2_N_2_	4.7	476.2	1.60%	0.1	93.90%	4.41	4.50%	0.21	1
	5.8	587.7	1.30%		95.10%	5.5	3.60%		
16_ex_CO_2_N_2_O_2_	5.8	587.7	1.30%	0.1	77.70%	4.5	21.00%	1.22	1

**Table 2:** Time-Domain HRV Features.

Feature	Description	Interpretation
AE	Approximate entropy	Measure of irregularity in HRV signal
AR_1	AR(2) model parameter a1	Reflects the HRV signal dynamics
AR_2	AR(2) model parameter a2	Reflects the HRV signal dynamics
AR_noise_var	Normalized variance of innovation signal	Reflects the overall HRV variations
meanHR	mean Heart rate	Represents ANS activity
meanNN	mean NN interval	Represents ANS activity
NN50count	Number of pairs of adjacent NN intervals differing by more than 50 ms in the entire recording (NN50 count)	Estimate of short-term HRV
NN50count1	Number of pairs of adjacent NN intervals differing by more than 50 ms in the entire recording (NN50 count)	Estimate of short-term HRV
NN50count2	Number of pairs of adjacent NN intervals differing by more than 50 ms in the entire recording (NN50 count)	Estimate of short-term HRV
pNN50	NN50 count divided by the total number of all NN intervals (pNN50)	Estimate of short-term HRV
r_RR	correlation coefficients of the lagged Poincaré plot	
RMSSD	root mean square of the successive difference	Estimate of short-term HRV
SD1	width of Poincaré plot	Reflects the short-term variability
SD2	length of Poincaré plot	Reflects short-term and long-term variablity
SDNN	standard deviation of normal-to-normal R peaks	Reflects overall variability in HRV

**Table 3:** HRV Power Spectral Density Features.

Feature	Description	Interpretation
HF	Power of high-frequency band (0.15–0.4 Hz)	Reflects parasympathetic activity
HFnu	Normalized units of HF power (0.15–0.4 Hz)	Reflects parasympathetic activity
LF	Power of low-frequency band (0.04–0.15 Hz)	Reflects both sympathetic and parasympathetic activity (more sympathetic)
LF_HF	LF–HF ratio	The ratio of sympathetic to parasympathetic activity
LFnu	Normalized units of LF power (0.04–0.15 Hz)	Reflects both sympathetic and parasympathetic activity (more sympathetic)
Power ratio	Ratio of power in frequency band (f < 0.6) to frequency band (f > 0.6)	Reflects spreading of the spectrum, mainly due to exercise
total_psd	total power	
VLF	Power of very-low-frequency band (<0.04 Hz) (VLF)	

**Table 4:** HRV Principal Dynamic Mode Features.

Feature	Description	Interpretation
PSymp	Parasympathetic	parasympathetic (non-linear calculation)
Symp	Sympathetic	sympathetic (non-linear calculation)

**Table 5:** Repeated-measures linear regression between each of the below-listed features and stage impairment scores.

	Effect Estimate (95% CI)	*p*-Value
Intercept	−0.14 (−0.51, 0.24)	0.4592
Exercise	0.33 (0.18, 0.49)	<0.0001
High N_2_	0.72 (0.55, 0.89)	<0.0001
High CO_2_	0.56 (0.4, 0.71)	<0.0001
High O_2_	0.21 (0.06, 0.37)	0.0070
Age	0 (−0.01, 0.01)	0.7845
Diving experience	0.06 (−0.13, 0.24)	0.5505
Video games experience	0.02 (−0.09, 0.12)	0.7721
Order (reversed vs. standard)	0.04 (−0.17, 0.24)	0.7114

**Table 6:** Repeated-measures linear regression interactions between each of the below-listed features and stage impairment scores.

	Degrees of Freedom	F Value	*p*-Value
Exercise	72	41.59	<0.0001
High N_2_	33	76.74	<0.0001
High CO_2_	71	60.51	<0.0001
High O_2_	70	14.42	0.0003
exercise*high_N_2_	33	14.3	0.0006
exercise*high_CO_2_	71	21.09	<0.0001
high_N_2_*high_CO_2_	32	1.34	0.2558
exercise*high_N_2_*high_CO_2_	32	4.66	0.0386
exercise*high_O_2_	70	9.86	0.0025
high_N_2_*high_O_2_	19	4.10	0.0572
exercise*high_N_2_*high_O_2_	19	0.97	0.3380
high_CO_2_*high_O_2_	70	0.46	0.4987
exercise*high_CO_2_*high_O_2_	70	0.19	0.6607
high_N_2_*high_CO_2_*high_O_2_	19	3.99	0.0604
exercise*high_N_2_*high_CO_2_*high_O_2_	19	0.00	0.9885

**Table 7:** Repeated-measures linear regression between each of the below-listed features and stage impairment scores.

	Effect Estimate (95% CI)	*p*-Value
HFnu	0.161 (0.062, 0.261)	0.0016
NN50count1	0.026 (0.001, 0.052)	0.0404
SDNN	0.099 (0.000, 0.198)	0.0500
NN50count2	0.013 (0.000, 0.026)	0.0524
SD2	0.108 (−0.003, 0.219)	0.0569
AR_2	0.374 (−0.111, 0.858)	0.1306
HF	0.054 (−0.019, 0.127)	0.1432
LF	−0.114 (−0.267, 0.04)	0.1467
SD1	0.023 (−0.012, 0.058)	0.2038
RMSSD	0.021 (−0.012, 0.054)	0.2081
total_psd	−0.189 (−0.519, 0.141)	0.2612
LFnu	−0.113 (−0.321, 0.095)	0.2875
AE	−0.114 (−0.344, 0.117)	0.3339
meanNN	0.377 (−0.456, 1.21)	0.3739
AR_1	−0.264 (−0.926, 0.397)	0.4325
meanHR	−0.256 (−0.904, 0.392)	0.4377
Symp	0.076 (−0.149, 0.3)	0.5068
PSymp	0.077 (−0.169, 0.322)	0.5384
VLF	0.022 (−0.066, 0.111)	0.6188
NN50count	0.002 (−0.007, 0.012)	0.6237
PowerRatio	0.014 (−0.067, 0.096)	0.7268
AR_noise_var	−0.003 (−0.033, 0.027)	0.8595
pNN50	0.001 (−0.009, 0.01)	0.8966
r_RR	0.001 (−0.019, 0.021)	0.9245
LF_HF	0 (−0.04, 0.039)	0.9842

**Table 8:** Repeated-measures linear regression between each of the below listed-features and stage impairment scores restricted to non-exercise stages.

	Effect Estimate (95% CI)	*p*-Value
RMSSD	0.095 (0.037, 0.153)	0.0015
SD1	0.095 (0.036, 0.154)	0.0017
NN50count1	0.034 (0.013, 0.056)	0.0019
NN50count2	0.016 (0.005, 0.026)	0.0049
SDNN	0.145 (0.038, 0.252)	0.0082
SD2	0.149 (0.021, 0.276)	0.0228
AR_1	−1.735 (−3.319, −0.152)	0.0319
AR_2	−0.705 (−1.621, 0.211)	0.1304
VLF	0.07 (−0.036, 0.176)	0.1933
total_psd	0.266 (−0.183, 0.715)	0.2434
r_RR	−0.109 (−0.302, 0.083)	0.2642
AE	−0.086 (−0.26, 0.088)	0.3313
AR_noise_var	0.019 (−0.022, 0.06)	0.3644
NN50count	0.004 (−0.005, 0.012)	0.3874
LFnu	−0.068 (−0.227, 0.092)	0.4034
PowerRatio	−0.031 (−0.135, 0.072)	0.5496
HFnu	−0.057 (−0.265, 0.15)	0.5878
pNN50	0.002 (−0.006, 0.009)	0.6971
meanNN	−0.157 (−0.957, 0.644)	0.7002
LF_HF	0.004 (−0.024, 0.033)	0.7601
meanHR	0.063 (−0.511, 0.638)	0.8279
HF	0.013 (−0.124, 0.151)	0.8467
Symp	−0.014 (−0.195, 0.167)	0.8771
PSymp	−0.014 (−0.214, 0.186)	0.892
LF	−0.009 (−0.156, 0.139)	0.9059

**Table 9:** Accuracies and F1 scores of machine-learning class assignments by MATB subtask.

Classifier	Accuracy	F1 Score
TRACKING (motor)	mean ± sd%	mean ± sd%
Random forest	81.29 ± 12.65%	66.63 ± 17.96%
Kernel SVM	79.88 ± 11.86%	66.86 ± 16.35%
MONITOR (attention)	mean ± sd%	mean ± sd%
Random forest	86.39 ± 9.18%	73.46 ± 16.23%
Kernel SVM	84.61 ± 10.4%	72.43 ± 16.76%
COMMUNICATIONS (memory)	mean ± sd%	mean ± sd%
Random forest	86.11 ± 9.11%	68.76 ± 17.84%
Kernel SVM	84.61 ± 10.4%	69.36 ± 16.5%
PUMP (strategy)	mean ± sd%	mean ± sd%
Random forest	81.11 ± 10.98%	70.4 ± 17.86%
Kernel SVM	77.05 ± 13.53%	68.93 ± 16.31%

**Table 10:** Machine learning regression values.

Regressor	Average R-Squared Value	Average Testing RMSE
SVM with RBF kernel	0.7699	0.987

**Table 11:** Classification for resting and exercising stages.

Classifier	Average Accuracy	Average F1 Score
**Resting stages**		
Random Forest	58.26%	33.37%
Kernel SVM	59.94%	**43.46%**
Linear SVM	**61.82%**	36.74%
KNN	56.26%	36.34%
**Exercise Stages**		
Random Forest	62.62%	65.42%
Kernel SVM	**67.14%**	**71.35%**
Linear SVM	61.90%	67.20%
KNN	61.67%	63.21%

**Table 12:** Pearson correlation: HRV feature score with stage impairment score (rest and exercise).

	Pearson Correlation	Sig. (2-Tailed)	N
Stage impairment score	1		432
AE	0.147 **	0.003	415
AR_1	−0.298 **	0	415
AR_2	−0.206 **	0	415
AR_noise_var	0.255 **	0	415
meanHR	0.271 **	0	415
meanNN	−0.297 **	0	415
NN50count	−0.003	0.946	405
NN50count1	0.024	0.636	382
NN50count2	0.048	0.332	405
pNN50	−0.021	0.668	405
r_RR	−0.059	0.233	415
RMSSD	0.049	0.316	415
SD1	0.049	0.322	415
SD2	−0.042	0.395	415
SDNN	−0.014	0.777	415
HF	0.024	0.662	341
HFnu	0.047	0.384	341
LF	0.028	0.61	341
LF_HF	0.045	0.411	341
LFnu	0.066	0.223	341
PowerRatio	−0.224 **	0	415
total_psd	−0.221 **	0	341
VLF	0.06	0.27	341
PSymp	−0.092	0.09	341
Symp	−0.083	0.126	341

** denotes statistical significance.

## Data Availability

Data are contained within the article.
